# Enhancement of the bioactive compounds and biological activities of maca (*Lepidium meyenii*) via solid-state fermentation with *Rhizopus oligosporus*

**DOI:** 10.1007/s10068-023-01508-6

**Published:** 2024-02-12

**Authors:** Kyeong Min Ryu, Hayeong Kim, Jiho Woo, Juho Lim, Choon Gil Kang, Seung Wook Kim, Taeyoon Kim, Doman Kim

**Affiliations:** 1 Graduate School of International Agricultural Technology, Seoul National University, Pyeongchang-gun, Gangwon-do 25354 Republic of Korea; 2 Institute of Food Industrialization, Institutes of Green Bioscience & Technology, Center for Food and Bioconvergence, Seoul National University, Pyeongchang-gun, Gangwon-do 25354 Republic of Korea; 3 Ottogi Corporation, Anyang-si, Gyeonggi-do 14060 Republic of Korea; 4 Fervere Campus Corporation, Pyeongchang-gun, Gangwon-do 25354 Republic of Korea

**Keywords:** Maca, *Rhizopus oligosporus*, Macamide B, l-Carnitine, Fermentation

## Abstract

Maca (*Lepidium meyenii* Walp) is renowned for its phytochemicals, including amino acids, saponins, and macamides, confer nutritional and medicinal benefits. This study analyzed the bioactive constituents of maca via solid-state fermentation with *Rhizopus oligosporus* for 0–15 days. After fermentation, the l-carnitine content reached 157.3 μg/g. A 93% increase in macamide B was recorded after 7-day fermentation. Total flavonoid and saponin contents increased by 88.2% and 110.3%, respectively. The fermentation process significantly enhanced the physicochemical attributes of maca; in particular, its water retention and cholesterol-binding capacities increased by 1.73- and 4.30-fold, respectively, compared with the non-fermented maca. Moreover, fermented maca exhibited stronger antioxidant and α-glucosidase-inhibiting effects than non-fermented maca. Finally, the neuroprotective effect of maca on HT-22 cells increased by 23% after 5-day fermentation. These findings demonstrate the potential of fermented maca as a novel ingredient for foods, beverages, and pharmaceuticals.

**Supplementary Information:**

The online version contains supplementary material available at 10.1007/s10068-023-01508-6.

## Introduction

Maca (*Lepidium meyenii* Walp) is a cruciferous vegetable primarily cultivated for over 2000 years in the Peruvian Andes that is consumed mainly as a nutritional supplement in capsule or powder form. The nutritional value of maca is similar to cereal grains but higher compared to carrots, potatoes, and turnip (da Silva Leitao Peres et al., 2020). The dry maca contains 8.9–11.6% proteins, 1.1–2.2% lipids, 8.2–9.1% fiber, 5.0% ash, and 54.6–60% carbohydrates. Moreover, maca contains secondary metabolites, including macamides, macaenes, polysaccharides, fatty acids, glucosinolates, alkaloids, and flavonols that contribute to its neuroprotective, anti-fatigue, antioxidant, antitumor, anti-inflammatory activity, sexual improvement, and fertility enhancement (Li et al., 2017; Lin et al., 2023; Carvalho and Ribeiro, 2019). Macamides, the main bioactive compounds found in maca, are composed of fatty acids and benzylamine varying in hydrocarbon chain length and degree of unsaturation. Currently, 26 macamides have been identified in maca (Chen et al., 2017). *N*-benzyl-hexadecanamide or macamide B is one of the most critical macamides mainly used as a biomarker for maca (Wu et al., 2013). Additionally, macamide B can inhibit fatty acid amide hydrolase, modulating anandamide expression (Wu et al., 2013). Furthermore, recent studies have highlighted that macamide B possessed neuroprotective effects, including its capacity to inhibit inflammatory cytokines and alleviate oxidative stress in PC12 cells (Yu et al., 2020).

Solid-state fermentation (SSF) has gained attention due to its simplicity, low sterilization cost, and high production concentration compared to submerged fermentation (Jiménez-Quero et al., 2020). SSF is the process of microorganisms growing on solid materials with a low water concentration (Hölker et al., 2004). SSF has been successfully used to produce biofuels, flavors, bioactive compounds, lipids, and enzymes (Sala et al., 2019). Our previous study revealed that solid-state fermentation (SSF) of quinoa, wild-turmeric, and ginseng leaves by *Rhizopus oligosporus* increased l-carnitine levels and enhanced biological activities, including antioxidant and anti-lipid accumulation effects (Hur et al., 2018; Lim et al., , 2022, 2023). *R. oligosporus*, the dominant fungus in fermented soybean products such as tempeh, produces various enzymes (e.g., amylase, protease, β-glucosidase, and β-glucuronidase), which contribute to the synthesis of γ-aminobutyric acid, polyphenols, and l-carnitine during fermentation (Vattem and Shetty, 2002). Moreover, *R. oligosporus* is generally recognized as a safe species because it does not produce toxic secondary metabolites (Londono-Hernandez et al., 2017). Although submerged fermentation of maca using *Lactobacillus* spp showed enhancement of inhibition activity against NO release LPS-stimulated RAW264.7 cells, melanin formation and tyrosinase activity in B16F10, fermentation of maca using SSF still limited. Therefore, this study aims to investigate the effects of SSF on maca using *R. oligosporus* on physicochemical characteristics and biological activities of maca. Macamide B, l-carnitine, ergosterol, total polyphenols, flavonoids, saponins, volatile compounds, and amino acids in fermented maca were analyzed. Furthermore, the physicochemical properties of fermented maca, such as water-holding capacity (WHC), water swelling capacity (WSC), oil absorption capacity (OAC), and cholesterol-binding capacity (CBC) were analyzed. The antioxidant activities via oxygen radical absorbance capacity, 2,2-diphenyl-2-picrylhydrazyl (DPPH) radical scavenging activity, and ferric reducing antioxidant power (FRAP), as well as α-glucosidase inhibition of fermented maca were measured. The neuroprotective effects of non-fermented and fermented maca on HT-22 cells were determined. Our findings demonstrate the potential benefits of maca fermented by *R. oligosporus* through SSF.

## Materials and methods

### Materials

Powdered maca was procured from Kapdang (Seoul, Korea). Macamide B, l-carnitine, and dimethyl sulfoxide were acquired from MedChemExpress (Monmouth Junction, NJ, USA), Tokyo Chemical Industry Co., Ltd. (Tokyo, Japan), and Sigma-Aldrich (St. Louis, MO, USA), respectively. Other chemicals acquired from Sigma-Aldrich included oleanolic acid, cholesterol, α-glucosidase (from *Saccharomyces cerevisiae*), and ergosterol. All other solvents and chemicals were of analytical grade.

### Fermentation of maca by *R. oligosporus* through SSF

#### Preparation of maca for fermentation

*R. oligosporus* KCCM 11948P, acquired during our previous study (Lim et al., 2023), was cultured on potato dextrose agar for 5 days at 30 °C. Maca powder was mixed with water at a ratio of 1:1.7 (w/v) for 12 h, then sterilized for 20 min at 121 °C. Subsequently, fermentation commenced at 30 °C with inoculating 5 × 10^5^ spores/g of maca powder. After 15 days, the fermented maca was stored lyophilized at − 45 °C and 10 Pa using the FD-550 instrument (Eyela, Tokyo, Japan).

#### Extraction of fermented maca

For extraction, 400 mg of non-fermented or fermented maca was added to a 100-mL beaker containing 57.8 mL of 100% (v/v) ethanol, followed by sonication using an ultrasonic homogenizer (KUS-1200; Korea Bio Tech, Seongnam, Korea). Subsequently, the mixture was maintained at 60 °C for 1 h, and the supernatant was obtained by centrifugation at 9,600×*g* for 15 min. Whatman No. 1 filter paper was used for filtration, and the entire process was repeated thrice. The ethanol in the sample was removed using an evaporation apparatus operating at 50 °C (Laborota 4000; Heidolph Instruments, Schwabach, Germany), and the sample was lyophilized at − 45 °C and 10 Pa for subsequent analyses.

### Analysis of phytochemical compounds in fermented maca

#### Analysis of l-Carnitine in fermented maca

To analyze the l-carnitine content of fermented maca, 20 mg of non-fermented or fermented maca was added to 1 mL dimethyl sulfoxide as stock. Subsequently, the sample was diluted using methanol and filtered through a 0.2-μm membrane syringe filter. l-carnitine analysis was conducted using ultrahigh performance liquidchromatography–mass spectrometry (UPLC-MS) with a QDa detector and a BEH HILIC column (2.1 × 100 mm, 1.7 μm; Waters, MA, USA) in accordance with our previous method (Lim et al., 2023). l-Carnitine (0.01–2.0 μg/mL) was used as standard.

#### Analysis of macamide B and ergosterol in fermented maca

Macamide B and ergosterol contents in non-fermented or fermented maca were analyzed by UPLC-MS using a BEH ${{\text{C}}}_{18}$ column (2.1 × 100 mm, 1.7 μm; Waters, Milford, MA, USA) with a photodiode array detector (Waters, Milford, MA, USA). Acetonitrile was used as a mobile phase to analyze macaminde B and ergosterol with isocratic elution for 10 min at a flow rate of 0.3 min/mL. Ergosterol and macamide B, ranging from 0.02 to 20.0 μg/mL, were used as standard.

#### Total phenolic content (TPC)

The Folin–Ciocalteu method measured the TPC of non-fermented or fermented maca, with 0–100 μg/mL gallic acid used for standard. TPC is expressed as mg gallic acid equivalent (GAE) per gram of maca (dry mass) (mg GAE/g DM).

#### Total flavonoid content (TFC)

The aluminum chloride colorimetric method measured the TFC of non-fermented or fermented maca, with 0–100 μg/mL quercetin used as standard. TFC is expressed as mg quercetin equivalent (QE) per gram of maca (dry mass) (mg QE/g DM).

#### Total saponin content (TSC)

The vanillin-sulfuric acid method was used to determine the TSC of non-fermented or fermented maca, with oleanolic acid used as standard. Briefly, non-fermented or fermented maca powders were mixed with an 8% vanillin (w/v) solution and 72% sulfuric acid (v/v) at a 1:1:10 (v/v/v) ratio. The mixtures were incubated at 60 °C for 10 min, then chilled for 5 min. Subsequently, the plate was read at 544 nm using a SpectraMax M3 microplate reader (Molecular Devices, Sunnyvale, CA, USA). TSC is expressed as mg oleanolic acid equivalent (OAE) per gram of maca (dry mass) (mgOAE/g DM).

#### Analysis of volatile profiles

Volatile profiles of non-fermented or fermented maca were analyzed using a gas chromatograph/mass selective detector (5975C TAD Series; Agilent, Santa Clara, CA, USA). Volatile profiles were obtained by headspace solid-phase microextraction (HS-SPME). Maca sample (1 g) was placed in 20-mL vials and subjected to HS-SPME at 60 °C for 10 min. The samples were then extracted by gas chromatography–SPME for 20 min. Helium was used as the transporter gas at a flow rate of 1.5 mL/min. The initial oven temperature was maintained at 50 °C for 3 min, raised to 150 °C at a rate of 5 °C/min, followed by an increase to 200 °C at a rate of 10 °C/min, and then an increase to 280 °C at a rate of 50 °C/min. Finally, the temperature was maintained at 280 °C for 3 min. The mass detection range was m/z 33–500.

#### Analysis of amino acids in fermented maca

To extract amino acids from fermented maca, 100 mg of each sample was hydrolyzed with 6 N HCl. The HCl was removed from the extracted sample by rotary evaporation at 55 °C. The resulting sample was dissolved in 0.02 N HCl and filtered through a membrane syringe filter (0.2 μm). Amino acid compounds were measured using an amino acid analyzer (L-8800; Hitachi, Tokyo, Japan) in accordance with the manufacturer's standard protocol.

### Analysis of antioxidant capacity

#### Ferric reducing antioxidant power assay

To conduct the FRAP assay, 20 μL of sample or ferrous sulfate heptahydrate (1–2000 μM) were mixed with 180 μL of FRAP solution containing 20 mM anhydrous ferric chloride, 10 mM 2,4,6-tripyridyl-s-triazine (TPTZ), and 0.3 M sodium acetate buffer (pH 3.6) at a 1:1:10 (v/v/v) ratio in a 96-well plate. After incubation for 30 min in the dark, the plate was recorded at 593 nm using a SpectraMax M3 microplate reader. FRAP is expressed as mg Fe^2+^ per gram of maca (dry mass) mM Fe^2+^/mg DM.

#### Oxygen radical absorbance capacity assay

To conduct the ORAC assay, 10 μL of sample or Trolox (1–50 μM) were mixed with 90 μL of fluorescein for use as a probe in a 96-well plate. Then, 100 μL of 2,2′-azobis(2-amidinopropane) dihydrochloride (AAPH) was added to initiate the reaction and induce radical formation. Fluorescence was measured every 3 min (λ_emission_ = 538 nm, λ_excitation_ = 485 nm) over 120 min at 37 °C using a SpectraMax M3 microplate reader. The net area under the curve (AUC) was calculated by subtracting the AUC of the blank from the AUC of the reacted sample. ORAC is expressed as mM Trolox equivalent (TE) per gram of maca (dry mass) (mM TE/g DM).

#### DPPH radical scavenging activity assay

For the DPPH assay, 100 μL of the sample was mixed with 80 μL of methanol in a 96-well plate. Subsequently, the reaction was initiated with 20 μL of 1 mM DPPH methanol solution. The reaction without DPPH was used as the negative control, and the reaction with DPPH and without the sample was used as the positive control. The mixture was reacted at 28 °C for 30 min, followed by reading at 517 nm using a SpectraMax M3 microplate reader.

### α-Glucosidase inhibition assay

For the α-glucosidase inhibition assay, various concentrations of non-fermented or fermented maca powder were added to reaction mixture compounds of 0.2 U α-glucosidase/mL in 50 mM potassium phosphate buffer (pH 6.8) and incubated at 37 °C for 5 min. The reaction was started by adding 5 mM *ρ*-nitrophenyl α-d-glucopyranoside and incubated for 8 min. Then, 250 mM Na_2_CO_3_ was added at a 1:1 (v/v) ratio to stop the reaction, and the reaction was measured at 405 nm using a SpectraMax M3 microplate reader.

### Physical characterization

The WHC, WSC, OAC, and CBC of non-fermented or fermented maca were analyzed using a previously described method with minor modifications (Guan et al., 2023; Luo et al., 2018).

#### Water holding capacity assay

To measure the WHC, 50 mg of fermented maca were hydrated with 1 mL of deionized water at room temperature for 24 h. Then, the supernatant was discarded by centrifugation at 10,000×*g* for 15 min, and the residue was collected and weighed. The WHC was calculated as follows:

$$\mathrm{WHC }({\text{g}}/{\text{g}}) = ({w}_{2}-{w}_{1})/{w}_{1},$$

where ${w}_{2}$ is the residue weight and ${w}_{1}$ is the dry sample weight.

#### Water swelling capacity assay

To measure the WSC, 50 mg of fermented maca were hydrated with 1 mL of deionized water at 4 °C for 24 h. The final volume of the swollen sample was measured. WSC was calculated as follows:

$$\mathrm{WSC }({\text{mL}}/{\text{g}}) = ({v}_{2}-{v}_{1})/{w}_{1},$$

where ${v}_{2}$ is the wet sample volume, ${v}_{1}$ is the dry sample volume, and ${w}_{1}$ is the dry sample weight.

#### Oil adsorption capacity assay

To measure the OAC, 50 mg of fermented maca were hydrated with 1 mL of olive oil at room temperature for 24 h. The supernatant was discarded by centrifugation at 10,000×*g* for 15 min, and the residue weight was determined. The OAC was measured as follows:

$$\mathrm{OAC }({\text{g}}/{\text{g}}) = ({w}_{3}-{w}_{1})/{w}_{1},$$

where ${w}_{3}$ is the residue weight and ${w}_{1}$ is the dry sample weight.

#### Cholesterol binding capacity assay

For sample preparation, 200 mg of fermented maca was added to 5 mL of 10% (v/v) egg yolk in water at pH 2.0 or 7.0. The mixture was incubated at 37 °C and 120 rpm for 2 h. The solution was subsequently mixed with four volumes of 95% ethanol and centrifuged at 3800×*g* for 20 min, and the supernatant was collected. Ethanol in the extracted sample was removed by evaporation. To measure CBC, the collected sample was mixed with glacial acetic acid, 0.05% (w/v) o-phthalaldehyde, and sulfuric acid at a ratio of 1:1:1:5 (v/v/v/v) and incubated for 20 min at 25 °C. Then, the reaction was read at 550 nm using a SpectraMax M3 microplate reader. CBC is expressed as milligrams per gram (mg/g).

### Cell viability test

HT-22 mouse hippocampal neuronal cells were cultured in Dulbecco’s modified Eagle’s medium (DMEM) containing 10% (v/v) fetal bovine serum, 100 µg/mL streptomycin, and 100 U/mL penicillin at 37 °C with 5% CO_2_ for 24 h until 70–80% confluency. Cells seeded in 96-well plates at 6 × 10^3^ cells/well and incubated at 37 °C with 5% CO_2_ for 24 h. Then, cells were treated with various concentrations of non-fermented or fermented maca (125–1000 μg/mL). After 24 h incubation at 37 °C with 5% CO_2_, 10% (v/v) Ez-Cytox solution in DMEM was added to each well, followed by incubation for 1 h. Then, the plate was read at 450 nm. Cell viability was estimated relative to the control.

### Neuroprotective effects

Neuroprotective effects of non-fermented and fermented maca were investigated using the HT-22 mouse hippocampal neuronal cell line. HT-22 mouse hippocampal neuronal cell at 6 × 10^3^ cells/well in a 96-well plate was treated with non-fermented or fermented maca (200 μg) and hydrogen peroxide (800 µM) and incubated for 18 h at 37 °C with 5% CO_2_. Cells treated with hydrogen peroxide (800 µM) were used as the positive control, while cells treated with DMEM were used as negative control. Then, 10% (v/v) Ez-Cytox solution in DMEM was added to each well, followed by incubation for 1 h. The plate was read at 450 nm. Neuroprotection effects were estimated relative to the negative control.

### Statistical analysis

All data are presented as means ± standard deviations (SD) for triplicate experiments. Group differences in fermentation time were tested by a one-way analysis of variance, followed by Duncan’s test and Pearson correlation analysis. All statistical analyses were performed using SPSS software (version 26.0; IBM Corp, Armonk, NY, USA). Graphs were generated using Prism 8.0 software (GraphPad Software Inc., San Diego, CA, USA).

## Results and discussion

### Effects of fermentation on the phytochemical characteristics of maca

#### Ergosterol and l-Carnitine contents of fermented maca

**Fig. 1:**
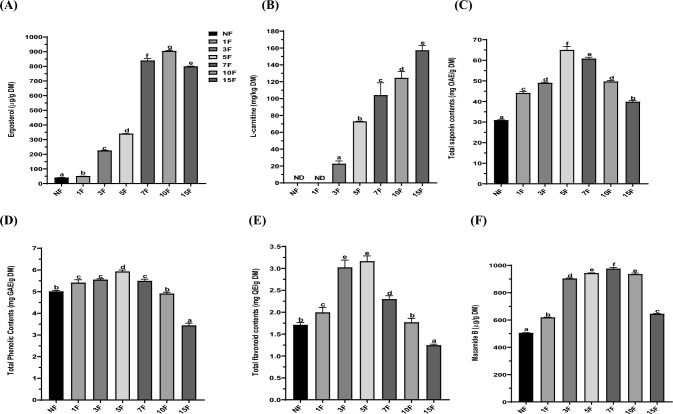
The ergosterol content (**A**), l-carnitine content (**B**), total saponin contents (**C**), total phenolic contents (**D**), total flavonoid contents (**E**), and macamide B content (**F**) of fermented maca by *R. oligosporus*. NF: non-fermented maca; 1F, 3F, 5F, 7F, 10F, and 15F: 1-day, 3-day, 5-day, 7-day, 10, and 15-day fermentation of maca, respectively. Data are means ± SD of three independent experiments. Different letters indicate statistical difference by Duncan’s test

The extraction yields of non-fermented and 1-, 3-, 5-, 7-, 10-, and 15-day fermented maca powders were 36.4 ± 1.2%, 36.3 ± 3.1%, 36.0 ± 4.3%, 36.0 ± 4.8%, 35.1 ± 3.1%, 35.34 ± 2.8%, and 34.7 ± 2.6%, respectively. Ergosterol, a 5,7-diene oxysterol, is a primary sterol in most fungal cell membranes. Ergosterol regulates cation permeability, cell growth, and membrane fluidity. There is a significant correlation between ergosterol content and fungal growth during fermentation because of its association with hyphal length (Ng et al., 2008). Moreover, ergosterol exhibits antitumor, antioxidant, and cholesterol-lowering effects (Yongxia et al., 2020). As shown in Fig. 1A, the ergosterol content increased from 40.6 ± 1.1 μg/g in non-fermented maca to 904.3 ± 3.3 μg/g at 10-day of fermentation; it subsequently decreased to 799.4 ± 2.2 μg/g after 15-day of fermentation 1-Carnitine is less contained in plant-derived foods than in animal-derived foods. It is a crucial quaternary amine that transfers long-chain fatty acids to mitochondria for β-oxidation (Park et al., 2017). Herein, l-carnitine was newly synthesized in maca by fermentation using *R. oligosporus*. The l-carnitine content was not detected in non-fermented and 1-day fermented maca. However, it was obtained at 22.67 ± 3.33 mg/kg DM at 3-day fermentation and reached 157.32 ± 5.57 mg/kg DM at 15-day fermentation (Fig. 1B). Increased fermentation time resulted in increasing l-carnitine content. A strong correlation was observed between l-carnitine and ergosterol content (r = 0.942). Our results were consistent with wild-stimulated ginseng leaves and wild turmeric fermentation using *R. oligosporus* that the l-carnitine contents in wild-simulated ginseng leaves and wild turmeric were not detected at 0-day fermentation and reached 242 mg/kg, and 119.0 mg/kg at 7-day fermentation, respectively (Lim et al., 2022, 2023). l-Carnitine was integrated from methionine and lysine by secreted trimethyllysine hydrolase, trimethyl-aminobutylraldehyde dehydrogenase, hydroxyl-trimethyllysine aldolase, methyltransferase, and trimethyllysine hydroxylase from *R. oligosporus* during fermentation (Rousta et al., 2021). The leucine and methionine contents in maca were 54.3- and 28.0 mg/g protein, respectively (Valerio and Gonzales 2005). Therefore, l-carnitine in fermented maca was synthesized from leucine and methionine in maca by *R. oligosporus* enzymes secretin during fermentation.

### Characterization of phytochemical compounds in fermented maca

#### TSC, TPC, and TFC

Maca contains many bioactive compounds, including phenolic compounds, flavonoids, and saponin. As shown in Fig. 1C, the TSC increased from 30.9 ± 0.2 mg OAE/g to 65.0 ± 1.8 mg OAE/g after 5 days of fermentation, then gradually declined to 39.9 ± 0.5 mg OAE/g after 15 days of fermentation. Meanwhile, the TPC increased from 5.0 ± 0.1 mg GAE/g in non-fermented maca to 5.9 ± 0.1 mg GAE/g after 5 days of fermentation; it subsequently decreased to 3.4 ± 0.1 mg GAE/g after 15 days (Fig. 1D). The TFC in maca rose from 1.7 ± 0.1 mg QE/g to 3.2 ± 0.1 mg QE/g after a 5-day fermentation period, then gradually declined to 1.3 ± 0.1 μg QE/g after 15 days (Fig. 1E). The contents of saponin, phenolic compounds, and flavonoids were 110.3%, 18.0%, and 88.2% higher, respectively, than the contents in non-fermented maca after 5 days of fermentation. The increases in TSC, TPC, and TFC in fermented maca can be attributed to the carbohydrate-cleaving enzymes produced by *R. oligosporus* during fermentation, which degrade the cell wall matrix (Lim et al., 2022). Regarding the significant increase in saponin content, saponin glucoside degrades to saponin aglycone due to the β-glucosidase produced during fermentation (Toor et al., 2021).

#### Macamide B contents of fermented maca

Macamides are bioactive compounds unique to maca. Among 26 detectable macamides in maca, macamide B, the predominant compound, indicates maca quality (Wu et al., 2013). Thus, the effects of fermentation on macamide B content of maca was analyzed using UPLC-MS. Figure 1F shows the fluctuations in macamide B content of maca throughout fermentation. The content of macamide B in maca increased up to 7 days of fermentation, then decreased for up to 15 days. The initial content of macamide B in non-fermented maca was 504.8 ± 2.6 μg/g. After 7 days of fermentation, the content rose to 975.2 ± 9.9 μg/g, representing an increase of 93.2% compared with non-fermented maca.

#### Changes in maca volatile profiles after fermentation

The volatile profiles of non-fermented and fermented maca are presented in Table 1. These compounds are commonly associated with maca and other substrates fermented using *R. oligosporus*. Consistent with the results of a previous study, benzyl cyanide, a degradation product of benzyl glucosinolate, was the major volatile compound in non-fermented maca (Sun et al., 2018). As fermentation progressed, acetic acid, benzyl cyanide, and benzaldehyde levels decreased. In contrast, the levels of 2,3-butanediol and benzyl alcohol increased. Similar changes have been observed during coffee fermentation by *R. oligosporus* (Lee et al., 2016). Ethyl acetate content was highest in 3-day fermented maca. Compounds such as 2,3-butanediol, which has a fruity, buttery scent; benzyl alcohol, which has a floral scent; and ethyl acetate, which has a sweet and fruity scent, were significantly more abundant in fermented maca than in non-fermented maca (Xu et al., 2022). Acetic acid flavor is sour like vinegar, so SSF of maca with *R. oligosporus* can reduce the sour flavor. However, the effect of SSF on maca’s flavor requires further study. Acetic acid flavor is sour like vinegar, so SSF of maca with *R. oligosporus* can reduce the sour flavor. These findings suggest that the fermentation of maca by *R. oligosporus* could enhance its scent.

**Table 1:** Profile of the volatile compounds in fermented maca using *R. oligosporus*

Compound	Retention time	Fermented maca (day, % of total)						
		0	1	3	5	7	10	15
Ethanol	1.88		8.2	16.5	8.1	11.9	13.6	6.9
Dichloromethane	2.04	1.8	1.8		7.6	6.6	9.0	3.2
Ethyl actetate	2.33				9.0	6.3	7.1	1.5
Acetic acid	2.370	23.0	14.4	9.5	6.0			
1,1′-[Ethylidenebis(oxy)]bis[hexane]	2.73			0.5		1.5		
Isoamyl alcohol hexamethyl-cyclotrisiloxane	3.60			1.4				
Triethylene glycol	4.27							0.5
2,3-Butanediol	4.52			5.2	17.1	18.4	21.9	30.5
Butanoic acid	4.79	1.0						
*N*-ethyl-1,3-dithioisoindoline	5.00				1.1	1.1	1.3	
Benzaldehyde	8.93	9.0	1.2	2.0	1.5	2.2	2.1	3.2
Octamethyl-cyclotetrasiloxane	9.90	0.6		0.7	1.3	1.4	1.4	1.1
Benzenemethanol	11.47	N/D	1.7					
Benzyl alcohol	11.52			6.09	9.98	9.1	16.7	17.7
2-Methyl-octane	13.28						1.5	
3-Methyl-eicosane	13.42					0.8	0.9	
Nonadecane	13.70				0.4	2.0		
Benzyl cyanide (benzyl nitrile)	14.25	48.0	70.6	40.2	20.3	14.6	8.8	6.7
Benzonitrile	14.52	3.8					0.7	
Benzyl isocyanide	14.63	3.8						
2-Methyl-benzonitrile	14.95			2.6				
Dodecane	15.79	0.6						
Dodecamethyl-cyclohexasiloxane	19.24				0.4	0.3		1.7
1,3,5-Triethyl-benzene	20.82				0.8	0.7	0.6	
*Trans*-caryophyllene	21.68				2.8	2.3	1.9	0.9
7,11-Dimethyl-3-methylene-1,6,10-Dodecatriene	22.56							0.6
1-(1,5-Dimethyl-4-hexenyl)-4-methyl-benzene	23.22							1.1
1-Methyl-3-(3,4-dimethoxyphenyl)-6,7-dimethoxyisochromene	23.49			1.3	2.6	2.2	1.8	1.0
5-Isopropenyl-3,6-dimethyl-6-ethenyl-4,5,6,7-tetrahydro-transbenzofuran	23.57							0.7
Butylated hydroxytoluene	23.87		0.7	0.8	1.4	1.2	0.9	0.8
Beta-bisabolene	24.73							1.6
2-Methyl-1-(1,1-dimethylethyl)-2-methylpropanoic acid	25.43	1.3		0.5	1.8	2.0	1.8	
Curzerenone	25.64							2.7
Longifolene-(V4)	26.09							1.1
1,3,5,7-Tetraethyl-1-ethylbutoxysiloxycyclotetrasiloxane	26.46	0.6	0.4	0.6	1.1	1.4	1.1	1.1
Italicene	26.65							5.4
7-Amino-1,4-dimethylpyrimido[4,5-c]pyridazine-3,5-(1H,2H)-dione	26.83					1.6		
Germacrone	27.04							0.7
2,5-Dimethylanisole	28.05	0.4		0.3	0.3	0.3		0.7
octadecamethyl-cyclononasiloxane	28.44	0.4		0.5	0.6	0.7	0.6	0.5
tetrakis (dimethylsilylcarbodiimide)	28.67	0.99						
1,2-Benzenedicarboxylic acid, butyl 2-methylpropyl ester	28.87	0.7	0.6	0.8	1.1	0.9	0.6	1.1
Eicosane	28.98	1.7		0.3	0.3	0.3		
1,2,9,10-Tetramethoxy-6-methyl-5,6,6a,7-tetrahydro-4H-dibenzo[de,g]quinoline	29.18				0.4	0.4	0.4	
1,1,3,3,5,5,7,7,9,9,11,11-dodecamethylhexasiloxane	29.38		0.1		0.4	0.5		
Dibutyl phthalate	29.42			1.5	0.4	0.5		0.2
Ethyl ester hexadecenoic acid	29.50				0.4	0.9	0.6	
6-Aza-5,7,12,14-tetrathiapentacene	29.96	0.4	0.3	0.9	0.6	2.0	0.8	0.4
2-(4-Methylphenyl)-Indolizine	30.05					0.4		
2,3-Dimethyl-4-azaphenanthrene	30.23			1.8				
hexamethyl-cyclotrisiloxane	30.31				0.2			1.1
3-Methyl-5-diphenyldihydrafuran	30.46	0.4		1.7		1.3		
Decamethyl-tetrasiloxane	30.70			1.6			0.5	
1,1,1,3,5,5,5-Heptamethyltrisiloxane	30.95	0.5		1.4	1.4	3.0	3.1	0.8
Gibberellin A3	31.65	1.0		0.5	0.7	1.7		

*ND* not determined

#### Changes in amino acids after fermentation

Amino acids are crucial substrates for synthesizing many substances and serve as building blocks for tissue proteins. They also offer various health benefits, including antioxidant properties (Zhang and Zhang, 2018). Among the many amino acids, only 20 serve as building blocks for proteins. Because essential amino acids cannot be synthesized in the human body, they must be ingested to satisfy nutritional requirements. The levels of 16 amino acids in non-fermented and fermented maca are detailed in Table 2. Eight essential amino acids (threonine, valine, lysine, leucine, isoleucine, methionine, phenylalanine, and histidine) were highest in 7-day fermented maca.

**Table 2:** Amino acids of fermented maca using *R. oligosporus*

Compound	Fermented maca (day, mg/g)						
	0	1	3	5	7	10	15
Aspartic acid	6.29	5.92	7.19	9.17	9.48	9.65	7.45
Threonine	3.00	2.91	3.58	4.28	4.30	4.12	3.12
Serine	2.89	2.83	3.50	4.18	4.23	4.11	3.14
Glutamic acid	6.84	7.41	10.14	11.06	10.74	10.51	8.95
Glycine	3.48	3.31	3.96	4.67	4.71	4.68	3.77
Alanine	4.71	4.16	5.74	7.37	7.41	6.78	4.97
Cystine	0.18	0.20	0.38	0.85	1.10	1.36	1.52
Valine	4.07	3.98	4.93	6.33	6.75	6.75	5.54
Methionine	0.48	0.50	0.75	1.31	1.34	1.32	1.25
Isoleucine	2.94	2.87	3.63	4.47	4.48	4.26	3.06
Leucine	4.52	4.40	5.57	6.89	6.98	6.60	4.72
Tyrosine	0.73	0.76	1.06	1.57	1.50	1.53	1.05
Phenylalanine	2.86	2.73	3.40	4.25	4.31	4.17	3.10
Lysine	3.33	3.11	4.39	5.75	5.78	5.41	3.69
Ammonia	1.65	1.56	2.11	3.74	5.45	6.76	10.37
Histidine	1.63	1.52	1.87	2.30	2.33	2.30	1.78
Arginine	7.28	6.94	6.16	5.60	5.25	4.81	3.36
Hydroxy proline	0.80	0.75	0.69	1.00	1.09	1.14	1.21
Proline	40.89	38.70	24.27	13.01	10.15	9.58	7.78

### Antioxidant capacity of non-fermented and fermented maca

**Fig. 2:**
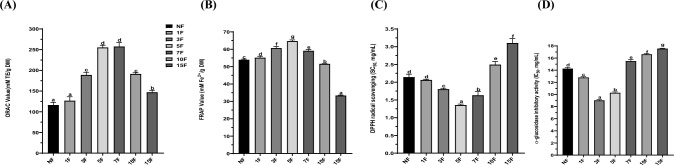
Antioxidant capacity via oxygen radical absorbance capacity (ORAC) (**A**), ferric reducing antioxidant power (FRAP) (**B**), 2,2-diphenyl-2-picrylhydrazyl (DPPH) radical scavenging activity (**C**), and $\alpha$-glucosidase inhibitory effect (**D**) of fermented maca by *R. oligosporus*. Data are means ± SD of three independent experiments. Different letters indicate statistical difference by Duncan’s test

Reactive oxygen species (ROS) are produced during mitochondrial oxidative metabolism. Oxidative stress arises from an imbalance caused by excessive ROS or oxidants relative to the cell’s ability to establish an effective antioxidant response (Liang and Kitts, 2014). This stress is linked to various diseases, including diabetes, neurodegenerative diseases, and aging. Therefore, maintaining proper ROS balance is essential for preventing various diseases caused by oxidative stress. Herein, the ORAC assay to assess the hydrogen atom transfer mechanism, the FRAP assay to investigate the single-electron transfer mechanism, and the DPPH assay to assess both mechanisms were used to evaluate the antioxidant capacities of non-fermented and fermented maca (Liang and Kitts, 2014). As shown in Fig. 2A, ORAC increased from 116.1 ± 6.7 mM TE/g in non-fermented maca to 257.1 ± 9.6 mM TE/g in 7-day fermented maca, then decreased to 146.9 ± 5.0 mM TE/g in 15-day fermented maca. The ORAC of 7-day fermented maca was 121.45% higher than that of non-fermented maca. However, ORAC did not significantly differ between the 5- and 7-day fermented maca. FRAP increased from 53.9 ± 0.5 mg Fe^2+^/g in non-fermented maca to 64.6 ± 0.7 mg Fe^2+^/g in 5-day fermented maca, then decreased to 33.3 ± 0.3 mg Fe^2+^/g in 15-day fermented maca (Fig. 2B). FRAP for the 5-day fermented maca was 19.85% higher than FRAP for non-fermented maca. The 50% DPPH scavenging concentration (SC_50_) rose from 2.14 ± 0.08 mg/mL in non-fermented maca to 1.35 ± 0.03 mg/mL in the 5-day fermented maca, then declined to 3.10 ± 0.13 mg/mL in the 15-day fermented maca (Fig. 2C) The DPPH ${{\text{SC}}}_{50}$ for the 5-day fermented maca was 36.8% higher than the DPPH SC_50_ for non-fermented maca. Because saponin and phenolic compounds possess potent antioxidant activity, they influence the overall antioxidant effects of fermented maca. Pearson correlation analysis was conducted to determine the relationships of total phenolic, and saponin contents with the antioxidant properties of maca. The TPC of fermented maca was strongly correlated with both FRAP (r = 0.992) and DPPH (r = 0.931). In contrast, the TSC exhibited a robust correlation with ORAC (r = 0.952). The antioxidant activities in non-fermented and fermented maca are attributed to the presence of phenolic and saponin, which have also been identified in wild turmeric and wild ginseng leaf (Lim et al., 2022, 2023).

### Analysis of α-glucosidase inhibition by fermented maca

Recently, diabetes has emerged as a key public health problem. α-Glucosidase is an essential enzyme that catalyzes the hydrolysis of oligo- and disaccharides, subsequently converting them into glucose. The inhibition of α-glucosidase can limit glucose absorption in the small intestine, thereby preventing diabetes (Proença et al., 2017). Thus, the inhibitory activity of non-fermented and fermented maca was determined via the half-maximal inhibitory concentration (IC_50_ value) against α-glucosidase activity. The IC_50_ declined from 14.25 ± 0.23 mg/mL in non-fermented maca to 9.01 ± 0.14 mg/mL in 3-day fermented maca, then rose to 17.54 ± 0.09 mg/mL in 15-day fermented maca (Fig. 2D). Pearson correlation analysis was performed to determine the relationship between TFC and α-glucosidase inhibition; a strong correlation was found (r = 0.863).

### Physicochemical properties of fermented maca

#### Effects of maca fermentation on WHC, WSC, and OAC

**Fig. 3:**
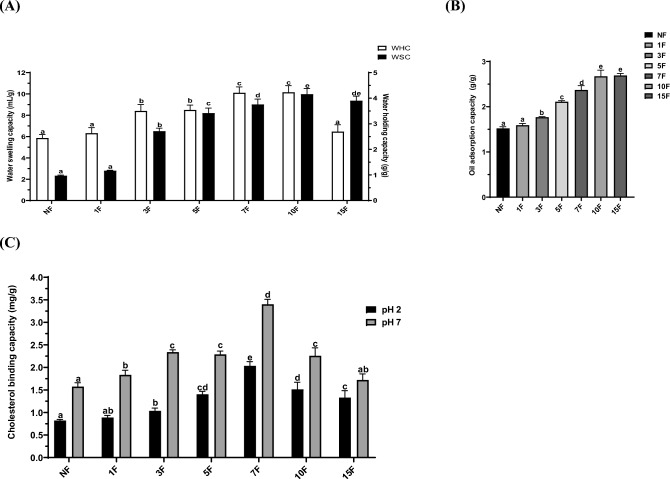
Physicochemical properties via water holding capacity (WHC), water swelling capacity (WSC) (**A**), oil adsorption capacity (OAC) (**B**), and cholesterol binding capacity (CBC) (**C**) of fermented maca by *R. oligosporus*. Data are means ± SD of three independent experiments. Different letters indicate statistical difference by Duncan’s test

The WHC, WSC, and OAC are vital properties in food processing. As shown in Fig. 3A, the WHC and WSC were increased in 10-day fermented maca, and both of these properties are important for hydration. Exposure to hydrophilic groups during the fermentation process and the amount of space within the molecular structure are factors that influence the WHC and WSC (Ma et al., 2023). Furthermore, the WHC is related to the disruption of glycosidic bonds and degradation of polysaccharides. This degradation process increases the storage space for water molecules and can occur through fermentation. The OAC is related to the absorption of organic compounds, which can facilitate the removal of excess fat during digestion. The OAC is increased in 10-day fermented maca (Fig. 3B); this increase is closely associated with the porous structure of the fiber, which is related to the adsorption of organic compounds onto the surface (Jeddou et al., 2016). These findings are consistent with a previous study of black rice fermented using *Neurospora crassa* (Guan et al., 2023).

#### Cholesterol binding capacity

Excessive cholesterol absorption and accumulation in the body can lead to coronary heart disease, obesity, and other ailments. CBC at pH values of 2.0 and 7.0 of non-fermented and fermented maca, representing the pH conditions in the stomach and small intestine, respectively, was evaluated. As shown in Fig. 3C, the highest CBC of maca at pH values of 2.0 and 7.0 were 2.03 ± 0.09 and 3.40 ± 0.11 mg/g on 7-day fermentation, respectively. The CBC was higher at pH 7.0 than at pH 2.0. A strong correlation was observed between CBC and saponin content in the fermented maca at pH 2.0 (*r* = 0.702) and 7.0 (*r* = 0.771). These results are consistent with previous fermented black rice findings (Guan et al., 2023). Evidence shows saponin regulates serum cholesterol levels (Vinarova et al., 2015). Saponins can form large, insoluble complexes with cholesterol, resulting in a cholesterol-lowering effect (Sharmin et al., 2021; Vinarova et al., 2015).

### Neuroprotective effects of fermented maca

**Fig. 4:**
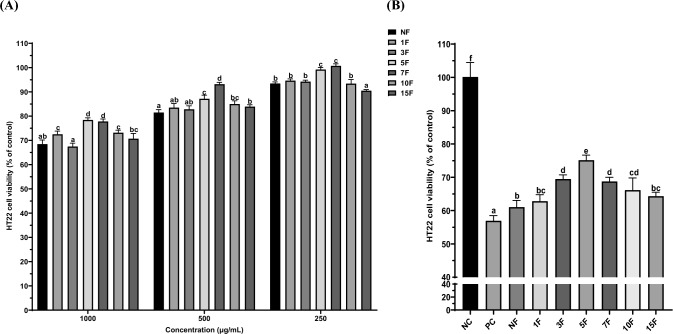
Cell viability (**A**) and neuroprotection effect (**B**) on HT-22 cells by non-fermented and fermented maca by *R. oligosporus*. Data are means ± SD of three independent experiments. Different letters indicate statistical difference by Duncan’s test

ROS can induce mitochondrial dysfunction and lipid peroxidation, leading to neuronal death, and oxidative stress is closely linked to neuronal diseases. Thus, the viability of HT-22 cells exposed to H_2_O_2_ is commonly used to assess neuroprotective effects during exposure to oxidative stress (Xu et al., 2013). As shown in Fig. 4A, non-fermented and fermented maca maintained over 80% cell viability at 250 and 500 μg/mL. Therefore, HT-22 cells exposed to H_2_O_2_ were treated with 200 μg/mL of non-fermented and fermented maca. The neuroprotective effect was highest after 5 days of fermentation and exhibited strong correlations with the flavonoid (*r* = 0.830), saponin (*r* = 0.900), and macamide B (*r* = 0.822) contents of fermented maca.

This study evaluated the effects of maca fermented by *R. oligosporus* through SSF. The TPC, TFC, TSC, macamide B, and ergosterol contents significantly increased during fermentation. Moreover, l-carnitine was synthesized. The increased levels of these compounds were associated with altered functional properties. Fermented maca exhibited higher antioxidant and α-glucosidase-inhibiting activities and stronger cholesterol-lowering and neuroprotective effects. Additionally, fermentation increased the essential amino acid contents. Our findings suggest that the fermentation of maca can significantly enhance its biochemical and physicochemical effects, positioning it as a valuable ingredient for beverages, foods, cosmeceuticals, and pharmaceuticals.

### Supplementary Information

Below is the link to the electronic supplementary material.

Supplementary file1 (DOCX 17 KB)
