# Supplementary material for: Enhancement of the bioactive compounds and biological activities of maca (Lepidium meyenii) via solid-state fermentation with Rhizopus oligosporus
Source: Food Sci Biotechnol. 2024 Feb 12;33(11):2585–96. doi: 10.1007/s10068-023-01508-6 (PMC11319679; doi:10.1007/s10068-023-01508-6)
Supplement: Supplementary file 1 — Supplementary file1 (DOCX 17 KB) [file 10068_2023_1508_MOESM1_ESM.docx]

**Supplementary materials**

**Enhancement of the Bioactive Compounds and Biological Activities of Maca (*Lepidium meyenii*) via Solid-State Fermentation with *Rhizopus oligosporus***

**Table S1. UPLC-PDA condition for standard sample**

**Table S2. UPLC-QDa mass condition for standard sample**

**Table S1. UPLC-PDA condition for standard sample**

| **Compound** | **Concentration range**  **(μg/mL)** | **Absorbance**  **(nm)** | **Linearity**  **(R^2)^** | **Regression equation** |
| --- | --- | --- | --- | --- |
| **Ergosterol** | **0.02 – 20.0** | **280** | **0.999** | **Y=51500x-11500** |
| **Macamide B** | **0.02 – 20.0** | **195** | **0.999** | **Y=160000x+1890** |

**Table S2. UPLC-QDa mass condition for standard sample**

| **Compound** | **Concentration range**  **(μg/mL)** | **M/Z** | **Polarity** | **Corn voltage**  **(V)** | **Capillary voltage**  **(kV)** | **Linearity**  **(R^2)^** | **Regression equation** |
| --- | --- | --- | --- | --- | --- | --- | --- |
| **L-carnitine** | **0.01 – 2.0** | **162.0** | **Positive** | **10** | **1.5** | **0.997** | **Y=9350000X+166000** |
